# The associates of anxiety among lung cancer patients: Dehydroepiandrosterone (DHEA) as a potential biomarker

**DOI:** 10.1186/s12885-024-12195-9

**Published:** 2024-04-15

**Authors:** Jia-Rong Wu, Vincent Chin -Hung Chen, Yu-Hung Fang, Ching-Chuan Hsieh, Shu-I Wu

**Affiliations:** 1 Department of Psychiatry, Chang Gung Memorial Hospital, 6, Sec. West Chia-Pu Road, 613 Pu-Zi City, Chiayi County Taiwan; 2 School of Medicine, Chang Gung University, 259 Wen-Hwa 1st Road, Kwei-Shan Tao-Yuan, Taiwan; 3 Division of Thoracic Oncology, Department of Pulmonary and Critical Care Medicine, Chang Gung Memorial Hospital, 6, Sec. West Chia-Pu Road, 613 Pu-Zi City, Chiayi County Taiwan; 4 Graduate Institute of Clinical Medical Sciences, College of Medicine, Chang-Gung University, 259 Wen-Hwa 1st Road, Kwei-Shan Tao-Yuan, Taiwan; 5 Department of Surgery, Chang-Gung Memorial Hospital, 6, Sec. West Chia-Pu Road, 613 Pu-Zi City, Chiayi County Taiwan; 6 Department of Medicine, Mackay Medical College, No.46, Sec.3, Zhongzheng Rd., Sanzhi Dist, 25245 New Taipei City, Taiwan; 7 Department of Psychiatry, Mackay Memorial Hospital, No. 92, Sec. 2, Zhongshan N. Rd., Zhongshan Dist., 104 Taipei City, Taiwan

**Keywords:** Anxiety, Lung cancer (LC), Dehydroepiandrosterone (DHEA), Chemotherapy

## Abstract

**Objective:**

Anxiety is a prevalent comorbidity in lung cancer (LC) patients associated with a decline in quality of life. Dehydroepiandrosterone (DHEA), a neuroactive steroid, levels rise in response to stress. Prior research on the association between DHEA and anxiety has yielded contradictory results and no study has investigated this association in LC patients.

**Methods:**

A total of 213 patients with LC were recruited from a general hospital. Data on demographic and cancer-related variables were collected. Using the Chinese version of the Hospital Anxiety and Depression Scale (HADS), the degree of anxiety was determined. Cortisol, DHEA, and Dehydroepiandrosterone sulfate (DHEA-S) levels in saliva were measured. Adjusting for confounding variables, a multivariate regression analysis was conducted.

**Results:**

147 men and 66 women comprised our group with an average age of 63.75 years. After accounting for demographic and treatment-related factors, anxiety levels were significantly correlated with, post-traumatic stress symptoms (PTSSs) (β = 0.332, *p* < 0.001) and fatigue (β = 0.247, *p* = 0.02). Association between anxiety and three factors, including DHEA, PTSSs, and fatigue, was observed in patients with advanced cancer stages (III and IV) (DHEA β = 0.319, *p* = 0.004; PTSS β = 0.396, *p* = 0.001; fatigue β = 0.289, *p* = 0.027) and those undergoing chemotherapy (DHEA β = 0.346, *p* = 0.001; PTSS β = 0.407, *p* = 0.001; fatigue β = 0.326, *p* = 0.011).

**Conclusions:**

The association between anxiety and DHEA remained positive in advanced cancer stages and chemotherapy patients. Further study is necessary to determine whether DHEA is a potential biomarker of anxiety in LC patients.

**Supplementary Information:**

The online version contains supplementary material available at 10.1186/s12885-024-12195-9.

## Introduction

### Lung cancer and anxiety

Lung cancer (LC) is the leading cancer-related cause of death, with an overall 5-year survival rate of only 10–20% worldwide [1]. LC is associated with not only physical symptoms but also increased psychological distress [2]. Anxiety and depression symptoms can negatively impact the quality of life and everyday function of patients with LC [3].

Pre- and post-cancer diagnosis, the prevalence of anxiety was substantially higher in individuals with LC than in the general population [4]. A recent study found that 43.5% of Chinese patients diagnosed with lung cancer experienced symptoms of anxiety [5]. Biological or behavioral factors may be the underlying causes of anxiety in people with LC. Anxiety is typically brought on by a patient’s psychological response to the diagnosis, treatment, relapse, end-of-life care, and survival [6]. A higher level of anxiety was associated with increased cancer-specific mortality [7] and lower quality of life in patients with LC [8]. Yet, anxiety is frequently underestimated and inadequately assessed in patients with LC, despite its significant impact on self-reported well-being [9].

Anxiety in cancer patients is associated with demographic variables such as sex [10], age [11], educational level [12], marital status [13], and work status [14]. Anxiety is likely connected to health conditions such as comorbid illnesses [12], psychiatric disorders [15], a history of alcohol consumption [16], and smoking [17]. Cancer characteristics, such as cancer stage [5] and treatment [18], are related to the onset of anxiety. Those diagnosed with LC at a young age, who did not undergo surgery, and who were undergoing radiotherapy have an elevated risk of anxiety [5].

### DHEA and anxiety

In addition to demographic and psychosocial characteristics, several biomarkers exist for detecting anxiety. Dehydroepiandrosterone (DHEA) is a neuroactive steroid that serves as a noncompetitive antagonist of the gamma-aminobutyric acid (GABA) receptor and a positive allosteric modulator of the N-methyl-D-aspartate (NMDA) receptor [19, 20]. DHEA and DHEA-S have been shown to be correlated with stress exposure [21, 22]. There was a positive association between DHEA concentration and anxiety, particularly in groups with a high level of anxiety, such as patients with generalized anxiety disorder and major depressive disorder [23]. However, few studies have investigated the link between anxiety and DHEA and DHEA-S in LC patients [24]. This study focused primarily on whether DHEA and DHEA-S levels may be potential biomarkers for anxiety in individuals with LC.

## Methods

### Participants

A cross-sectional study was conducted at the Oncology Outpatient Clinic of Chiayi Chang Gung Memorial Hospital. Between November 2017 and December 2020, 239 patients aged ≥ 20 years with biopsy-proven newly diagnosed primary LC were identified. We excluded patients whose Eastern Cooperative Oncology Group (ECOG) performance status was greater than 2 (i.e., ECOG = 3 to 4), those who lacked family support or lived alone, those who were unable to complete the questionnaires, those who did not provide informed consent, those with known brain metastases, and those with untreated obstructive sleep apnea. Individuals who met the inclusion criteria were recruited from the outpatient clinic by research assistants with a bachelor’s degree in nursing and extensive knowledge of psychiatry. Informed consent was obtained from all participants. This research involved 213 patients in all, and the refusal rate was 11%. Ethical approval was obtained from Institutional Review Board of Chiayi Chang Gung Memorial Hospital (201700297B0C106).

### Measures

#### Demographic data

We collected data on the following patient factors: sex, age, education level, marital status, employment status, history of smoking, history of alcohol drinking, psychiatric or physical illnesses (such as diabetes or hypertension), cancer stage, and treatments.

#### Endocrine assessment

Cortisol, DHEA, and DHEA-S levels in saliva were measured using the DHEA luminescence immunoassay. Saliva was collected into collection tubes, kept on ice immediately, and then stored at -30 °C until analysis. The activation of the hypothalamic–pituitary–adrenal (HPA) axis by physical and psychological stressors (i.e., anxiety) results in the release of HPA hormones, including cortisol, DHEA, and DHEA-S [25]. Cortisol and DHEA mediate physiological activities for homeostasis via their antagonistic biological actions, while DHEA-S and DHEA exert the same physiological effects [20].

#### Assessment of anxiety

The Chinese version of the Hospital Anxiety and Depression Scale (HADS) is a commonly utilized self-reported questionnaire for assessing depression and anxiety in Taiwanese cancer patients [26]. Two subscales (depression and anxiety) and seven items make up the HADS. Each item is rated between 0 (*not at all*) and 3, while the total subscale score spans from 0 to 21. The higher the score, the greater the distress. 0–7, 8–10, and 11–21 are the score ranges for noncases, borderline cases, and clinical cases, respectively. Strong concurrent validity of the HADS questionnaire was demonstrated by the association between its scales and subscales (i.e., depression and anxiety) and those of other frequently administered questionnaires (*r* = 0.49–0.83) [27]. The HADS is regarded as an indispensable self-assessment instrument for cancer patients [28], with fair validity and reliability in detection and monitoring [29].

#### Other psychological measurements

Cancer symptoms, such as the level of chronic pain [30], depression [24], fatigue [31], post-traumatic stress symptoms (PTSSs) [32], subjective cognitive impairment [33], and quality of life [34], and social factors, including family support [35], are psychological factors related to anxiety.

##### Chronic pain

The single-item visual assessment scale (VAS) is used to measure the intensity of pain, with scores ranging from 0 to 10. Greater scores indicate greater pain intensity. According to reports, this scale has satisfactory reliability and validity among Chinese patients [36].

##### Depression

The Patient Health Questionnaire (PHQ) is a self-administered questionnaire. Each of the nine criteria for depression is scored from “0” (*not at all*) to “3” (*nearly every day*). This questionnaire is frequently used to investigate mental disorders and is a reliable and valid instrument for determining the degree of depression [37]. The Chinese version of the PHQ had a Cronbach’s coefficient of 0.938% [38].

##### Cancer-related fatigue

The Brief Fatigue Inventory (BFI) is a screening instrument designed to measure cancer-related fatigue (CRF) severity [39]. This scale comprises nine items, each of which is graded on an 11-point scale. The first three questions assess the severity of fatigue, ranging from 0 (*no fatigue*) to 10 (*as bad as you can imagine*). The last six questions assess fatigue interference, ranging from 0 (*does not interfere*) to 10 (*completely interferes*). The Chinese version of this scale had an excellent Cronbach’s α reliability of 0.92 [40].

##### Post-traumatic stress symptoms (PTSSs)

A positive correlation exists between PTSSs and anxiety levels [32]. The English version of self-reported 4-item Startle, Physiological Arousal, Anger, and Numbness (SPAN) scale was first developed in 1999, and was derived from the previous 17-item Davidson Trauma Scale (DTS) [41]. Item selection for SPAN was drawn from 243 patients with post-traumatic stress disorder (PTSD) and sensitivity, specificity, positive predictive value (PPV), and negative predictive value (NPV) of items were calculated to determine the final 4 representative items [41]. Each score of items ranged from 0 (*not at all distressing*) to 4 (*extremely distressing*). For optimal sensitivity and specificity, the standard threshold was chosen at 5 [42], which suggests a higher risk of PTSD and greater severity of PTSSs [43]. The Chinese version of the SPAN scale (SPAN-C) was extracted from the Chinese version of DTS (DTS-C) and was first introduced in 2003 with excellent internal consistency (Cronbach’s α = 0.77) and reliability [44].

##### Cognition

The Functional Assessment of Cancer Therapy-Cognitive Function (FACT-cog), a self-administered questionnaire designed to evaluate perceived cognitive function and its impact on quality of life (QOL) in cancer survivors, was used to assess cognitive function. Its subscale consists of four dimensions and 33 items: perceived cognitive impairment (18 items), perceived cognitive capacities (7 items), the influence of reported cognitive impairment on quality of life (4 items), and comments from others on cognitive function (4 items). The response options range from 0 (*never*) to 4 (*several times a day*), with higher values indicating greater self-reported cognitive functioning [45]. This questionnaire has been extensively conducted on diverse cancer groups and verified in multiple languages [46, 47].

##### Quality of life

The EuroQol visual analog scale (EQ-VAS) is used to measure self-reported health conditions. Each item is scored between 100 (*best imaginable health state*) to 0 (*worst imaginable health state*) [48]. Both the English and Chinese versions of the scale showed great validity among cancer patients [49].

##### Family support

Gabriel Smilkstein established the family APGAR score in 1978 to evaluate family functioning. It is a 5-item questionnaire measuring family adaptation, partnership, growth, affection, and resolve. Each item is assigned a score between 0 (*hardly ever*) and 2 (*almost always*). A higher total score suggests greater family satisfaction. The coefficient for the family APGAR score was 0.85, showing favorable internal consistency [50]. The validity of the APGAR family questionnaire on family function was established by utilizing Family Function Index (FFI) and showed great validity and reliability [51].

### Statistical analysis

A Pearson’s correlation analysis was conducted to determine the relationship between continuous variables and anxiety level. A univariate study was performed to explore the link between the demographic characteristics of LC patients and their anxiety levels. Using a linear regression model, correlations between anxiety and continuous variables were determined. We undertook a multivariate linear regression analysis in which all significant correlations from the unadjusted model were included. The Bonferroni correction, a multiple-testing method, was utilized to adjust the *p*-value to mitigate the risk of type I error. Different treatments (with or without surgery and with or without chemotherapy) and cancer stages were analyzed by subgroup. In the multivariate regression model, adjusted *R*^2^ was utilized to measure the percentage of variation in dependent variables. The variance inflation factor (VIF), with a recommended level of < 5, was applied to examine the severity of multicollinearity. As a measure of association, regression coefficients (β value) are presented. Type I error level was set at 0.05. PASW Statistics 20.0 software was used for the analyses (IBM SPSS Inc., Armonk, NY, USA).

## Results

A total of 213 patients with LC were included in this study. Table 1 presents demographic characteristics and their association with anxiety. The majority of participants were male (57.9%). The anxiety levels of women were substantially higher than those of men (*p* = 0.037). A total of 27.2% of the participants possessed a high school diploma or a higher level of education, which exhibited a positive correlation with their anxiety levels (*p* = 0.01). The majority of participants were married and unemployed. Around half of the individuals had previously smoked. More than three-quarters of the individuals (76.8%) did not drink regularly. Fewer than 50% of the patients had mental or physical problems. Anxiety was significantly related to the co-occurrence of psychiatric illnesses and diabetes. About one-third of the participants were diagnosed with cancer in its early stages (37.6%), while the remaining patients had cancer in its advanced stages (62.4%). More than half of the patients were treated with chemotherapy, 42.7% with surgery, and 28.5% with radiation therapy. Table 1 shows that the mean age of the patients was 63.75 years (standard deviation = 9.75 years). Age was negatively associated with anxiety (*p* = 0.033). The average BMI was 24.46, with a standard deviation of 3.86. DHEA (*p* = 0.003), DHEA-S (*p* = 0.013), family support (*p* < 0.001), depression (*p* < 0.001), EQ-VAS scores (*p* < 0.001), CRF (BFI; *p* < 0.001), PTSSs (*p* < 0.001), and cognition (*p* < 0.001) were also strongly linked with anxiety (Table 2). Table 3 provides a summary of the significant univariate linear regression model predictors of anxiety in LC patients. After adjustment for significant variables and applying Bonferroni correction, the multivariate linear regression model revealed that PTSSs (β = 0.332, *p* < 0.001), and CRF (β = 0.247, *p* = 0.02) were independently associated with anxiety (Table 4). The adjusted *R*^2^ was 0.412 in this multivariate regression model, suggesting that 41.2% of the variance in anxiety was explained by PTSS, and CRF severity. The model’s VIF value was 1.204, which is less than O’Brien’s threshold value of 5 [52], indicating that there is no obvious multicollinearity between the variables.

**Table 1:** Univariate analysis of associations between patients with lung cancer characteristics and anxiety levels (HADS)

Characteristics	*N* = 213 (100%)		HADS scores	
			Mean (SD)	*p*-value
Gender				0.037*
Male	147 [69]		3.00 (3.87)	
Female	66 [31]		4.21 (3.94)	
Education				0.010*
Below senior high school	144 (67.6)		2.90 (3.52)	
Senior high school or higher	69 (32.4)		4.38 (4.51)	
Marital status				0.799
Married	186 (87.3)		3.35 (3.87)	
Not married, widowed, or divorced	27 (12.7)		3.56 (4.33)	
Employment				0.168
Full-time	33 (15.5)		4.24 (4.47)	
No	180 (84.5)		3.22 (3.80)	
Smoking				0.169
Never	90 (42.3)		3.96 (4.05)	
Past smoker	113 (53.1)		2.99 (3.77)	
Current smoker	10 (4.6)		2.50 (4.14)	
Drinking				0.665
Yes	18 (8.5)		4.33 (3.97)	
No	195 (91.5)		3.29 (3.91)	
Psychiatric history				0.001*
Yes	25 (11.7)		5.76 (4.78)	
No	188 (88.3)		3.06 (3.69)	
Diabetes				0.007*
Yes	44 (20.7)		2.32 (2.43)	
No	169 (79.3)		3.65 (4.19)	
Hypertension				0.981
Yes	87 (40.8)		3.37 (4.01)	
No	126 (59.2)		3.38 (3.87)	
Cancer stage				0.641
0-II	80 (37.6)		3.54 (3.67)	
III-IV	133 (62.4)		3.28 (4.07)	
*Systemic treatments*				
Surgical				0.900
Yes	89 (41.8)		3.34 (3.56)	
No	124 (58.2)		3.40 (4.17)	
Chemotherapy				0.835
Yes	130 [61]		3.33 (4.04)	
No	83 [39]		3.45 (3.74)	
Radiotherapy				0.362
Yes	60 (28.2)		2.98 (3.26)	
No	153 (71.8)		3.53 (4.15)	
Target therapy				0.993
Yes	50 (23.5)		3.38 (4.06)	
No	163 (76.5)		3.37 (3.89)	
HADS: Hospital Anxiety and Depression Scale				

*P value < 0.05 is statistically significant

**Table 2:** Pearson’s correlation of anxiety (HADS) and other psychological measures

Characteristics	HADS		
	Mean (SD)	r	*p*-value
Age (years-old)	63.75 (9.75)	-0.146	0.033*
BMI (kg/m^2^)	24.46 (3.86)	0.035	0.611
DHEA	92.42 (83.50)	0.286	0.003*
DHEA-S	2.35 (2.50)	0.239	0.013*
Cortisol	0.17 (0.12)	0.188	0.119
Family support (family APGAR score)	8.14 (2.75)	-0.273	< 0.001*
Chronic pain (score)	0.44 (0.50)	0.129	0.06
Depression (PHQ-9)	4.98 (4.91)	0.561	< 0.001*
EQ-VAS	66.69 (17.02)	-0.263	< 0.001*
CRF (BFI)	1.21 (1.77)	0.437	< 0.001*
PTSS (SPAN)	0.53 (1.58)	0.526	< 0.001*
FACT-Cog			
Perceived cognitive impairments	67.60 (6.89)	-0.345	< 0.001*
Comments from others	15.67 (1.18)	-0.146	0.033*
Perceived cognitive abilities	22.28 (5.22)	-0.101	0.143
Impact on quality of life	15.46 (1.72)	-0.304	< 0.001*
Total score	120.35 (11.87)	-0.295	< 0.001*

EQ-VAS: Quality of Life measures

*P value < 0.05 is statistically significant

**Table 3:** Summary of the significant predictors for anxiety in patients with lung cancer from the univariate linear regression model (*n* = 213)

Significant Predictors		Univariate analysis		
	β	95% CI	*P* value	R^2^
Characteristics				
Gender	-0.143	-0.281, -0.009	0.037	0.021
Age (year-old)	-0.146	-0.284, -0.012	0.033	0.021
Education	0.182	0.050, 0.326	0.01	0.031
Psychiatric history	0.222	0.09, 0.354	0.001	0.049
Diabetes	-0.138	-0.276, -0.004	0.037	0.014
Psychological measurements				
Family support	-0.273	-0.401, -0.141	< 0.001	0.074
Quality of life (EQ-VAS)	-0.263	-0.404, -0.136	< 0.001	0.069
Cognitive (FACT-Cog total score)	-0.295	-0.419, -0.164	< 0.001	0.087
Perceived cognitive impairments	-0.345	-0.456, -0.21	< 0.001	0.119
Comments from others	-0.146	-0.298, -0.012	0.033	0.021
Impact on quality of life	-0.304	-0.469, -0.189	< 0.001	0.092
Depression (PHQ-9)	0.561	0.439, 0.659	< 0.001	0.314
PTSS (SPAN)	0.526	0.39, 0.61	< 0.001	0.276
CRF (BFI)	0.437	0.315, 0.559	< 0.001	0.191
Endocrine assessment				
DHEA	0.286	0.099, 0.462	0.003	0.082
DHEA-S	0.239	0.05, 0.418	0.013	0.057

**Table 4:** Subgroup analysis and multivariate linear regression model for statistically significant anxiety-related variables in lung cancer patients

Significant variables of different groups	Multivariate analysis			
	Beta	*P* value	adjusted R^2^	VIF
LC patients			0.412	1.204
PTSSs	0.332	< 0.001		
CRF	0.247	0.02		
Subgroup analysis				
Chemotherapy				
Yes				
DHEA	0.346	0.001	0.451	1.255
PTSSs	0.407	0.001		
CRF	0.326	0.011		
No				
EQ-VAS	-0.605	0.001		
Surgery				
Yes				
DHEA	0.276	0.023	0.469	1.282
No				
DHEA	0.322	0.022		
CRF	0.374	0.017		
Cancer stage				
Early stage (0-II)		No significance		
Advanced stage (III-IV)				
DHEA	0.319	0.004	0.426	1.221
PTSSs	0.396	0.001		
CRF	0.289	0.027		

VIF: Variance Inflation Factor

### Subgroup analysis of patients receiving different treatments

The subgroup analysis included patients who received either chemotherapy or surgical intervention. Both treatments are regarded as standard options for treating patients with LC [53].

DHEA (β = 0.346, *p* = 0.001), PTSSs (β = 0.407, *p* = 0.001), and CRF (β = 0.326, *p* = 0.011) remained significantly associated with anxiety in the chemotherapy group among the adjusted predictors of anxiety (Table 4). The adjusted *R*^2^ was 0.451, indicating that 45.1% of the variance in anxiety was explained by DHEA, PTSS, and CRF. The VIF was 1.255, indicating the absence of multicollinearity. In the surgical subgroup, DHEA was positively associated with anxiety in both surgical (β = 0.276, *p* = 0.023) and non-surgical patients (β = 0.322, *p* = 0.022) (Table 4), indicating that DHEA has no predictive value for anxiety in LC patients who have undergone surgery.

### Early stage versus advanced stage

We conducted a subgroup analysis of anxiety-related factors in LC patients with early or advanced disease (Table 4). There was no significant association between DHEA and anxiety levels in patients with early-stage cancer.

Anxiety was positively correlated with DHEA (β = 0.319, *p* = 0.004), PTSSs (β = 0.396, *p* = 0.001), and CRF severity (β = 0.289, *p* = 0.027) in the advanced-stage cancer group (stages III and IV). The adjusted *R*^2^ was 0.426, indicating that DHEA, PTSS, and CRF explained 42.6% of the variance in anxiety. The VIF was 1.221, indicating that there was no obvious multicollinearity between the variables.

## Discussion

This study examined the association between various factors and anxiety in LC. Female sex, higher education level, psychiatric history, higher DHEA levels, higher DHEA-S levels, depression, severe CRF, and PTSSs were positively associated with anxiety, while older age, diabetes, poor family support, poor quality of life, and poor cognition were negatively associated with anxiety. The results of the multivariate model revealed that anxiety in patients with LC was significantly correlated with CRF and PTSSs. After adjusting for demographic and psychological variables, DHEA, CRF and PTSSs showed a positive correlation with anxiety in chemotherapy and advanced-stage disease subgroups.

In previous literature, anxiety in cancer patients was positively associated with female sex, younger age, lower educational level, CRF, depression, more comorbidities, more advanced cancer stage, those who received cancer treatment (chemotherapy, radiotherapy, surgery), and those with a previous history of mental health problems [9, 13, 54]. By contrast, we found that a higher educational level was positively correlated with anxiety. Possible explanations were that patients with higher educational levels might have a better understanding of poor prognosis and treatment-related discomforts; they may also encounter notable discrepancies in their physical and mental well-being upon receiving a diagnosis of LC.

### DHEA and anxiety

Previous studies examining the relationship between DHEA and anxiety have yielded contradictory results. Only certain groups of patients, such as those with major depressive disorder and generalized anxiety disorder exhibited a positive association [55]. The population of previous studies focused on adolescents, pregnant women, and healthy young adults [22, 23, 56], yet the association between DHEA and anxiety in cancer patients was not clearly established. In prior literature, DHEA was associated with depression and fatigue in Non-Small-Cell Lung Cancer After Chemotherapy, as well as cognitive dysfunction in breast cancer patients [57, 58]. To our knowledge, there were currently no research mentioning an association between anxiety and DHEA in cancer patients. In our study, DHEA was found to be significantly associated with anxiety levels in patients with LC undergoing chemotherapy and those with advanced-stage cancer. The majority of chemotherapy patients experienced severe anxiety [59] and patients with advanced LC are typically more anxious than those with early-stage LC [60]. Possible mechanism suggested that only at higher concentrations does DHEA negatively modulate GABAA receptors [55], which may reduce neuronal inhibition via GABA and exert anxiogenic effects [23]. Current evidence is primarily derived from cross-sectional studies, and possible mechanisms are inferred inductively from in vitro studies or animal models. Additional in vivo studies and longitudinal research examining the association between DHEA and anxiety are required.

### CRF and anxiety

Consistent with previous research, we found a strong correlation between anxiety and CRF [31]. This finding is comparable to that of previous research included patients with advanced LC and breast cancer receiving chemotherapy. Higher levels of anxiety are associated with increased CRF [61]. CRF is most severe in patients with LC, possibly due to the pulmonary system’s extensive functional impairment [59]. In this study, the association between CRF and anxiety was observed in chemotherapy and advanced disease subgroups; this finding is comparable to that of previous research [59, 62]. Neuroimmune and neuroinflammatory processes governed by the autonomic nervous system and HPA axis are potential mechanisms underlying the psychological impact of CRF [63]. Although the cellular inflammatory process has received considerable attention, the neural mechanism underlying the association between CRF and anxiety is poorly understood [63]. Future research should investigate this mechanism.

### PTSSs and anxiety

Our study revealed a positive correlation between PTSSs and anxiety in LC patients, especially in patients with advanced disease and patients undergoing chemotherapy. This finding is consistent with those of previous research, revealing a significant link between anxiety and PTSSs [64]. A single-centered cross-sectional study including 1017 cancer patients found a correlation coefficient of 0.708 between anxiety and PTSSs [65]. However, the study didn’t provide specific anticancer treatment, type of cancer, and stage, which resulted in different trauma and stress levels and different severities of PTSSs [65]. The advanced stage of cancer is associated with poor prognosis, and patients were likely to receive more treatment; both these factors impose considerable psychological burden (i.e., anxiety and PTSSs) and increase the risk of PTSD [66]. Chemotherapy patients are particularly susceptible to PTSSs and PTSD [67] due to the traumatic experiences caused by chemotherapy side effects and the treatment itself, which serves as a prolonged reminder of cancer [68]. Prior research focused on other types of cancer (breast, head and neck, brain, gynecological, and colorectal), whereas the evidence for LC is limited. Due to a lack of statistical power, a few studies have reported no significance [69]. Studies investigating the neurobiological mechanisms of PTSS in cancer patients have focused primarily on pediatric cancer survivors [70, 71]. Future research should place emphasis on adult patients.

Anxiety in cancer patients led to reduced quality of life, yet it was frequently overlooked and not properly assessed [9]. By using advanced biomarkers to detect anxiety early, we can provide immediate intervention for patients suffering from severe anxiety, improving adherence to cancer treatment and the overall health of patients [11].

### Strength

As per our knowledge, this is the first study to identify potential biomarkers and factors associated with anxiety in LC patients. Multiple psychological assessment instruments were utilized. To determine the factors associated with anxiety in the LC population, we evaluated PTSSs, family support and further investigated potential biomarkers such as cortisol, DHEA, DHEA-S, which were not typically evaluated in previous studies targeting cancer patients. We removed recruiting barriers by employing experienced research assistants in the field of psychiatry, using a well-defined protocol, obtaining informed consent, and ensuring good communication between research and clinical staff [72].

### Limitations

This research has several limitations. Due to the cross-sectional study design, it is challenging to infer a causal relationship between DHEA and anxiety. Future longitudinal studies should be conducted to reach a definitive conclusion. Second, because this was a single-center study, it lacked external validity for changes in clinical practice affecting a large population [73]. Third, patients may tend to conceal or exaggerate their anxiety symptoms when filling out the self-reported questionnaire, resulting in self-report and recall bias [74]. Integrating additional objective measures may reduce the risk of potential bias. Fourth, residual and unmeasured confounders could still exist because we were unable to collect all of the data. Future studies should consider incorporating socioeconomic status, cultural background, and comorbidities beyond diabetes and psychiatric illnesses (etc. obstructive sleep apnea) to gain a more comprehensive understanding of the patient population. Fifth, reverse causality may exist, such that anxiety may affect and be affected by DHEA levels [27]. It may be necessary to conduct larger-scale or multi-center prospective studies in the future to overcome these limitations.

## Conclusion

In this cross-sectional study, CRF and PTSS were found to be associated with anxiety in patients with LC. DHEA, CRF and PTSSs were significantly associated with anxiety in patients receiving chemotherapy and those with advanced cancer. This is the first study to report a correlation between DHEA levels and anxiety in individuals with LC. Numerous biomarkers are currently being investigated due to the growing interest in elucidating the biological basis of anxiety. Because anxiety in patients with LC is frequently underestimated, potential biomarkers, such as DHEA, would be useful for the early detection of anxiety in such populations. The focus of future research should be on the use of DHEA as a potential biomarker of anxiety in diverse cancer patient populations, and longitudinal changes in the association between DHEA and anxiety should be determined.

### Electronic supplementary material

Below is the link to the electronic supplementary material.

Supplementary Material 1

## Data Availability

All data generated or analyzed during this study are included in this published article and its supplementary information files.
